# The Mental Peculiarities and Disorders of Childhood

**Published:** 1860-06

**Authors:** Charles West


*The Mental Peculiarities and Disorders of Childhood.* By Charles West, M. D., etc. No one can have watched the sick-bed of a child without being struck by the almost unvarying patience with which its illness is borne, and the extremity of the peril from which, apparently in consequence of that patience, a complete recovery takes place.

No sorrow, gloomy foreboding, remorse, disappointment, nor anxiety, depresses the spirits and weakens the vital powers. Death, in general, brings no alarm. To keep the child happy, remove all causes of alarm, suffering, and discomfort; modify the treatment so as to escape a struggle with waywardness, and if death appears imminent, look at it from a child's point of view; all these are duties of the utmost importance both for physician and parents.

The mind of the child is feebler than that of an adult, but is proportionally active, and vivid in its imaginations. The child who dreads solitude, and asserts that it hears sounds or sees objects, often tells a literal truth. The sounds have been heard; in the stillness of the nursery, the little one has listened to what seemed to be a voice calling it; or, in the dark, phantoms have risen before its eyes, and the agony of terror betrays an impression far too real to be explained away, or to be unsuitably met by hard words or unkind treatment.

Disorder of the cerebral functions greatly exaggerates these impressions. Hence, the circumstances surrounding a child, whether in health or disease, are of vast importance, and should never be lost sight of in the treatment.

The passions, too, of a child, are exaggerated as a general rule. Reason as yet does not govern its caprices. There is also an intense craving for sympathy, which sometimes becomes quite uncontrollable, and requires as much care and judicious management as in the case of an adult monomaniac. As in diseases of the body, so in affections of the mind in early life, the power of repair causes a constant hope, which is not to be indulged in the cases of adults. Dullness, apathy, and cerebral disturbance, have, therefore, not so grave an import as at a more advanced age. The whole of the intellectual energy is expended on the child's commerce with the world; his relations to it are disturbed, hence, the want of interest, the slowness to resume the lively prattle after sickness should not be viewed with too much apprehension. The memory of a child is feeble, and when recovery takes place, it has to learn again its old lessons, and with weakened faculties. This process will extend over a longer time in proportion as it was younger at the time of the illness.

One thing should be remembered, in protracted illness, even when unaccompanied with disorder of the brain—the sense of hearing may be impaired, and this may be one cause of the child's dullness. The arrest of development, or the positive retrocession of the mental faculties, is of far less import than any perversion of the moral powers. The child who, in spite of dullness, manifests the ordinary childish feelings, may be much improved by judicious training. We must also, in forming an estimate of these capabilities, consider the accompaniments of the sickness. Convulsions or serious cerebral disturbance will correspondingly impair more profoundly the intellectual powers, and retard the recovery. It must be remembered that a very large number of children whose progress has been arrested at a very early age are allowed to grow up without any culture, and much of their dullness may be due to neglect. Apart from congenital instances, where mind and body are alike arrested in development, or are alike feeble and deformed, the state of the moral powers are more important as a guide to the prognosis than the condition of the intellectual. Want of affection, mischief, spite, causeless rage, are less hopeful than intellectual dullness, and the first step should be the establishment of moral control. It should be borne in mind that the heart may break or reason fail under causes seemingly insufficient, and the griefs of childhood may be as overwhelming as those of the strong man. The intellectual powers should never be overtasked. Thus may be laid the foundation of hydrocephalus, or the tubercular cachezia, the destruction of the nervous system or serious injury to the moral character.

Occasionally, children exaggerate their ailments, or feign those which have no existence, and they will put up with scanty fare and painful treatment as long as they can engross attention, and be the centre around which the household turns. —*Med. Times and Gazette*, Feb. 11, 1860.

